# Efavirenz and neuropsychiatric effects

**DOI:** 10.4102/sajhivmed.v18i1.741

**Published:** 2017-04-24

**Authors:** Mukesh Dheda

**Affiliations:** 1 Pharmacovigilance Centre for Public Health Programmes, National Department of Health, South Africa; 2 School of Health Science, University of KwaZulu-Natal, South Africa

Currently, efavirenz (EFV) is widely prescribed as part of antiretroviral therapy (ART) in South Africa, and it is most frequently prescribed in fixed-dose combination (FDC) at a dose of 600 mg.

Efavirenz has been linked to early (two to six weeks^1^) transient as well as late neuropsychiatric effects, which include increased risk of suicidal ideation,^2^ encephalopathy,^3^ catatonia,^4^ psychosis and ataxia.^5,6^ All of these have been directly linked to EFV toxicity. The risk for toxicity has been associated with loss of function polymorphisms of cytochrome 2B6, the main metabolising enzyme for EFV.^7^ It is estimated that about 20% of sub-Saharan Africans are genetically slow metabolisers and may be at risk of EFV toxicity.^8^

Clinicians should be aware that weight is another factor that predisposes a patient to EFV toxicity, and it is recommended that patients weighing less than 40 kg should be prescribed a reduced dose of 400 mg.^5,9^ There are no FDCs with a reduced EFV dose available in South Africa, and underweight patients are often over-dosed as healthcare workers often prescribe the FDC with standard doses of EFV.

Efavirenz toxicity should be considered in patients who present with depression, psychosis, catatonia, encephalopathy or ataxia after the first few weeks of therapy when other causes are excluded. These include renal failure, liver failure, vitamin B12 deficiency, syphilis, meningitis and structural brain lesions. Once suspected, it is recommended that EFV be switched to lopinavir or ritonavir. If patients are on first-line tuberculosis medication, the dose of lopinavir or ritonavir needs to be increased, and this can be done over two weeks if gastrointestinal tract (GIT) side effects are a problem.

Healthcare professionals are requested to be vigilant and report any suspected adverse drug reaction (ADR) when using antiretroviral (ARV) drugs. Please complete adverse drug reaction forms when adverse reactions are suspected.

Adverse drug reaction forms can be obtained from the National Department of Health Pharmacovigilance Centre for Public Health Programmes. Any comments and queries can be addressed to the email below:

Tel: +27 (0)12 395 9506/8099

Fax2email: 086 241 2473

Email: npc@health.gov.za
